# The lncRNA SOX2OT rs9839776 C>T Polymorphism Indicates Recurrent Miscarriage Susceptibility in a Southern Chinese Population

**DOI:** 10.1155/2019/9684703

**Published:** 2019-11-16

**Authors:** Zhenzhen Fang, Di Che, Shuang Qing, Qingfeng Li, Hui Men, Lianxiong Yuan, Li Li, Xiaoqiong Gu

**Affiliations:** ^1^Program of Molecular Medicine, Guangzhou Women and Children's Hospital, Zhongshan School of Medicine, Sun Yat-Sen University, Guangzhou, China; ^2^Department of Clinical Biological Resource Bank, Guangzhou Institute of Pediatrics, Guangzhou Women and Children's Medical Center, Guangzhou Medical University, Guangzhou, China; ^3^Department of Obstetrics and Gynecology, Guangzhou Women and Children's Medical Center, Guangzhou Medical University, 9 Jinsui Road, Guangzhou, Guangdong 510160, China; ^4^Department of Biostatistics, Sun Yat-Sen University, Guangzhou, China; ^5^Department of Blood Transfusion, Guangzhou Women and Children's Medical Center, Guangzhou Medical University, Guangzhou, China; ^6^Department of Clinical Laboratory, Guangzhou Women and Children's Medical Center, Guangzhou Medical University, Guangzhou, China

## Abstract

Genetic susceptibility may be involved in the onset of recurrent miscarriage. Previous studies have shown that some genetic polymorphisms that regulate cell migration are associated with susceptibility to recurrent miscarriage. The SOX2 overlapping transcript (SOX2OT) may regulate the migration and invasion of multiple tumor cells and is related to susceptibility to various diseases. However, whether lncRNA SOX2OT polymorphisms are related to recurrent miscarriage susceptibility is unclear. Therefore, we investigated the relationship between the lncRNA SOX2OT rs9839776 C>T polymorphism and recurrent miscarriage susceptibility. We recruited 570 subjects with recurrent miscarriage and 578 healthy control subjects from a population in southern China and used the TaqMan method for genotyping. We found a significant association between the rs9839776 CT genotype in the SOX2OT gene and an increased risk for recurrent miscarriage (CT vs CC: adjusted OR = 1.357, 95%CI = 1.065 − 1.728, *P* = 0.0134). However, we did not observe any significant associations between the recurrent miscarriage risk and the number of miscarriages in different age groups. In conclusion, our study indicated that the rs9839776 CT genotype may contribute to an increased risk of recurrent miscarriage in the southern Chinese population and that rs9839776 may act as a prognostic biomarker in recurrent miscarriage patients. However, an experiment-based study with a larger sample size should be performed to confirm these results.

## 1. Introduction

Recurrent miscarriage is defined as the loss of two or more pregnancies with an unknown etiology before the 20th week of gestation with the same male partner [1, 2]. Although the etiology is often unknown, studies have found that the migration function of trophoblasts is related to recurrent miscarriage [3, 4]. There are also research findings suggesting that recurrent miscarriage is associated with many genetic polymorphisms, including some genes, such as IGF-2 and PAI-1 that regulate cell migration [5–7]. Therefore, investigating the relationship between genetic polymorphisms that regulate cell migration, cell invasion, and recurrent miscarriage may help improve the understanding of the pathogenesis of recurrent miscarriage.

Long noncoding RNAs (lncRNAs) are defined as RNA transcript molecules (longer than 200 nucleotides) that are not translated into proteins [8, 9]. Recently, increasing research has shown that lncRNAs, such as regulators of transcription, tumorigenesis, cell migration, and invasion, are involved in numerous cellular and carcinogenesis processes [10–13]. Increasing research has confirmed that lncRNAs are involved in the occurrence and development of spontaneous miscarriage by regulating the migration and invasion of trophoblast cells [14, 15]. In addition, some studies have found that lncRNA polymorphisms affect the expression of lncRNAs [16, 17]. Moreover, research has shown that lncRNAs are associated with a number of diseases, such as breast cancer, cardiovascular disease, and recurrent miscarriage [18–20]. To date, multiple lncRNA polymorphisms, such as *lncRNA MALAT1* and *lncRNA CCAT2*, have been associated with susceptibility to recurrent miscarriage [20, 21]. Therefore, investigating the relationship between lncRNA gene polymorphisms and recurrent miscarriage can help to improve the understanding of the pathogenesis of recurrent miscarriage.

The SOX2 overlapping transcript (SOX2OT) is a lncRNA that is located in the SOX2 gene [22]. Recent studies have found that lncRNA SOX2OT acts as a carcinogenic molecule in the pathogenesis of many human cancers, such as gastric cancer, colorectal cancer, and breast cancer [23–26]. Research has found that SOX2OT may regulate the migration and invasion of multiple tumor cells, including non-small-cell lung cancer cells, hepatocellular carcinoma cells, gastric cancer cells, and colorectal cancer cells [25, 27–29]. Recently, a study by Tang et al. found that SOX2OT polymorphisms (rs9839776 C>T) are associated with breast cancer susceptibility via their influence on the expression of SOX2OT [22]. Moreover, a study conducted with a population in Tehran showed that breast cancer may be associated with reproductive risk factors [30]. Additionally, reproductive factors (e.g., abortions) constitute one type of risk factor for breast cancer, and abortion causes the mammary epithelium to proliferate and increases susceptibility to carcinogenesis [31–33]. Furthermore, our previous study found that some of the breast cancer susceptibility related lncRNA gene polymorphisms are also associated with susceptibility to recurrent miscarriage [21]. The abovementioned studies suggest that SOX2OT gene polymorphisms may be associated with recurrent miscarriage. To date, there has been no study on the effect of SOX2OT polymorphisms and the risk of recurrent miscarriage in a Chinese population. To better understand the potential association between SOX2OT polymorphisms and recurrent miscarriage, we conducted a case-control study to assess and quantify the association between rs9839776 C>T and the risk of recurrent miscarriage in a Chinese population.

## 2. Materials and Methods

### 2.1. Ethics Statement

This study was approved by the Medical Ethics Committee of Guangzhou Women and Children's Medical Center (Guangzhou, China, 2018022202). All female subjects who participated in this study signed and approved a written informed consent form.

### 2.2. Study Population

The study population consisted of a total of 570 patients with recurrent miscarriage (diagnosed as two or more spontaneous miscarriages of unknown etiology with the same male partner) and 578 pregnant female subjects with 2 or more normal pregnancies (no miscarriages) as controls.

### 2.3. DNA Extraction and SNP Genotyping

Genomic DNA was extracted from 200 *μ*L of peripheral blood leukocyte samples from all participants using the Blood DNA Isolation Kit (Tiangen, Beijing, China) according to the manufacturer's instructions. Specific single nucleotide polymorphism (SNP, rs9839776, C_42766292_10, catalog number: 4351379) genotyping fluorescent probes were purchased from ABI (Applied Biosystems TaqMan, Foster City, CA). SNP genotyping was performed on ABI Q6 instruments (Applied Biosystems TaqMan, Foster City, CA) with 384-well plates according to the TaqMan real-time polymerase chain reaction protocol. PCR amplification was performed in a final volume of 5 *μ*L, which included 0.04 *μ*L of primers, 2.5 *μ*L of 2x Master Mix (Tiangen, Beijing, China, catalog number: FP211), 1.46 *μ*L of ddH_2_O, and 2.5 ng of DNA.

### 2.4. Statistical Analysis

The data were entered into a database contained in SAS software (version 9.4; SAS Institute, Cary, NC, USA). Hardy-Weinberg equilibrium (HWE) for the control group and clinical and demographic characteristics of the two groups were compared using the goodness-of-fit *χ*^2^ test. In addition, the independent effects of each polymorphism on recurrent miscarriage risk were assessed by logistic regression analysis. An odds ratio (OR) with a 95% confidence interval (CI) was used to determine the association between polymorphisms and recurrent miscarriage. A *P* value ≤ 0.05 was considered statistically significant.

## 3. Results

### 3.1. Population Characteristics and SNP Selection

We recruited 570 patients with recurrent miscarriage between 20 and 46 years of age and 578 healthy control subjects between 20 and 49 years of age (Table 1). There were no significant differences in age between patients with recurrent miscarriage and the control subjects (32.63 ± 5.34 vs. 32.52 ± 5.42 years, *P* = 0.7278). In addition, approximately 55.26% of the patients with recurrent miscarriage experienced two or three miscarriages, and more than 44.74% of the patients experienced four or more miscarriages in this study.

### 3.2. The Relationship between the lncRNA SOX2OT rs9839776 C>T Polymorphism and Recurrent Miscarriage Susceptibility

The independent association between the SOX2OT genotype distribution of the SNP rs9839776 C>T and recurrent miscarriage risk was evaluated. The distribution of genotypes for rs9839776 C>T is shown in Table 2. The rs9839776 C>T polymorphism frequencies followed the Hardy-Weinberg equilibrium (*p*_HWE_ = 0.252). We found a significant correlation between the rs9839776 CT genotype and an increased risk of recurrent miscarriage (CT vs. CC: adjusted OR = 1.357, 95%CI = 1.065 − 1.728, *P* = 0.0134).

### 3.3. Stratified Analysis of Selected Polymorphisms (rs9839776) and Recurrent Miscarriage Susceptibility

A stratified analysis of subjects by age and the number of miscarriages further evaluated the effect of the SOX2OT rs9839776 C>T polymorphism in patients with recurrent miscarriage and control subjects (as shown in Table 3). The results showed that the SOX2OT rs9839776 C>T polymorphism was not significantly associated with recurrent miscarriage risk or the number of miscarriages in different age groups.

## 4. Discussion

In this case-control study with 570 cases and 578 controls, we evaluated the association of the SOX2OT rs9839776 C>T polymorphism with the risk of recurrent miscarriage in a southern Chinese population. We found a significant association between the rs9839776 CT genotype in the SOX2OT gene and an increased risk of recurrent miscarriage. However, we did not observe any significant association with the recurrent miscarriage risk or the number of miscarriages in different age groups. To the best of our knowledge, this is the first study to investigate the relationship between the lncRNA SOX2OT rs9839776 C>T polymorphism and susceptibility to recurrent miscarriage.

The SOX2OT gene, which is one of the major regulators of pluripotency, is located at the 3q26.3-q27 site of the human chromosome [34]. Studies have shown that lncRNAs can regulate the expression of adjacent antisense and overlapping genes through various mechanisms [35]. Because the intron region of SOX2OT contains the SOX2 gene, the SOX2 gene may be regulated by the SOX2OT gene. The Li et al. study found that together, Oct4 and Sox2 enhance the anti-inflammatory effects of human adipose tissue-derived mesenchymal stem cells [36]. Additionally, Spisek et al. found that SOX2 may be a target for specific immunotherapy in myeloma patients [37]. Previous studies suggest that the dysregulation of SOX2OT may play an important role in the inflammatory response by regulating SOX2 expression. In some somatic cancers, the dysregulation of SOX2OT expression and its concomitant expression with SOX2 has become a prominently observed phenomenon, and SOX2OT plays a key role in pluripotency and tumorigenesis [23]. Recent research has shown that the overexpression of SOX2OT is correlated with aggressive tumor behavior in gastric cancer [38]. Wang et al. found that lncRNA SOX2OT has carcinogenic effects, regulates the migration and invasion of osteosarcoma cells, and acts as a prognostic biomarker in osteosarcoma patients [39].

Research reports on SOX2OT gene polymorphisms are still few. rs9839776 is an intron variant in the SOX2OT gene [40], and many studies have found that rs9839776 in SOX2OT is associated with susceptibility to many diseases [22, 40]. A genome-wide association study by Boraska et al. found that rs9839776 in SOX2OT is likely associated with anorexia nervosa susceptibility [40]. Tang et al. found that the SOX2OT SNP rs9839776 is strongly associated with an increased risk of breast cancer and higher expression levels of SOX2OT, suggesting that rs9839776 enhances the onset of breast cancer by influencing the expression of SOX2OT [22]. Previous studies have shown that some genetic polymorphisms associated with breast cancer may also be associated with miscarriages [21, 41–46]. However, whether lncRNA SOX2OT polymorphisms are related to miscarriages has not been reported. In our present case-control study, we found that the rs9839776 CT genotype in SOX2OT may contribute to an increased risk of recurrent miscarriage. To the best of our knowledge, this is the first study to verify the relationship between the lncRNA SOX2OT rs9839776 CT genotype and the risk of recurrent miscarriage in southern China. Our results indicate that the rs9839776 CT genotype plays an important role in the pathogenesis of recurrent spontaneous miscarriages. The rs9839776 polymorphism in the SOX2OT gene is related to SOX2OT expression in breast cancer [22]. Furthermore, SOX2OT may regulate cell migration and invasion of multiple tumor cells [25, 27–29]. Moreover, trophoblast migration function plays an important role in the process of recurrent miscarriage. However, we do not have to detect the expression level of SOX2OT in recurrent miscarriage patients. We speculated that the rs9839776 polymorphism may increase the susceptibility to recurrent miscarriage by regulating the expression of SOX2OT. However, its specific molecular mechanism requires further research.

This study found that the number of miscarriages and the age of the patients at the time of pregnancy are risk factors for miscarriages. The risk of miscarriage was observed to increase sharply after the age of 35 from 9.5% in subjects 20-24 years of age to 76% in subjects aged 45 years and older. In clinical practice, females ≥ 40 years of age are less likely to become pregnant, and the risk of miscarriage increases [47, 48]. However, in this study, we did not observe any significant association between the recurrent miscarriage risk or the number of miscarriages in different age groups and rs9839776 in the SOX2OT gene.

The current research has some limitations. First, the analysis of miscarriages included the age of the subjects and the number of miscarriages, but due to a lack of information, other factors, such as alcohol consumption, smoking, and family history, which may affect the results of this study, were not considered in the stratified analysis. Second, the study was limited to the southern Chinese population and did not assess cases and controls of other ethnic groups. Considering the large difference in the incidence of miscarriages among different ethnic groups, the distribution and function of rs9839776 C>T in different populations require further investigation. Third, we did not analyze the expression level of SOX2OT. The relationship between the rs9839776 C>T polymorphism and its expression level requires further investigation.

In conclusion, this case-control study confirmed that the lncRNA SOX2OT rs9839776 CT genotype is associated with an increased susceptibility to recurrent miscarriage, but its risk effect was not different among female subjects of all ages, and there was no difference in the number of miscarriages. A larger sample size study and experiments should be performed to confirm the role of the lncRNA SOX2OT polymorphism in the susceptibility to recurrent miscarriage.

## Figures and Tables

**Table 1:** Frequency distribution of selected characteristics in recurrent miscarriage and control subjects.

Variables	Cases (*n* = 570)		Controls (*n* = 578)		*P* ^a^
	No.	%	No.	%	
Age range, years	20-46		20-49		
Mean ± SD	32.63 ± 5.34		32.52 ± 5.42		0.7278
**<**35	369	64.74	393	67.99	
35-40	147	25.79	125	21.63	
**>40**	54	9.47	60	10.38	
No. of abortions (%)					
2-3	315	55.26			
**≥**4	255	44.74			

^a^Two-sided *χ*^2^ test for distributions between recurrent miscarriage patients and controls.

**Table 2:** Genotype and allele frequencies of SOX2OT in RM patients and controls.

Genotype/allele	RM (*N* = 570)	Controls (*N* = 578)	*P* value^a^	OR (95% CI)	*P* value	Adjusted OR (95% CI)	*P* value^b^
SOX2OT/rs9839776 C>T (HWE = 0.252)							
CC	361 (63.33)	369 (63.84)		1.00	/	1.00	/
CT	182 (31.93)	180 (31.14)	/	**1.351 (1.061-1.721)**	**0.0146**	**1.357 (1.065-1.728)**	**0.0134**
TT	27 (4.74)	29 (5.02)	/	1.244 (0.726-2.133)	0.4268	1.247 (0.727-2.139)	0.422
Additive			0.9444	1.007 (0.826-1.228)	0.9474	1.007 (0.826-1.228)	0.9439
Dominant	209 (36.67)	209 (36.16)	0.8582	1.022 (0.804-1.300)	0.8582	1.024 (0.805-1.303)	0.8451
Recessive	543 (95.26)	549 (94.98)	0.8254	0.941 (0.550-1.611)	0.8255	0.935 (0.546-1.601)	0.8061

^a^ *χ* ^2^ test for genotype distributions between recurrent miscarriage patients and controls. ^b^Adjusted for age. RM: recurrent miscarriage.

**Table 3:** Stratification analysis for associations between SOX2OT polymorphisms and recurrent miscarriage risk in a south Chinese population.

Variable	rs9839776 (cases/controls)		*P*	OR (95% CI)	*P*	Adjust OR (95% CI)	*P* ^a^
	CC	CT/TT					
Age							
<35	236/248	133/145	0.807	0.964 (0.718-1.295)	0.8071	/	/
35-40	87/79	60/46	0.4982	1.184 (0.725-1.934)	0.4986	/	/
>40	38/42	16/18	0.9656	0.982 (0.440-2.195)	0.9656	/	/
No. of abortion (%)							
2-3	204/369	111/209	0.7838	0.961 (0.721-1.280)	0.7842	0.958 (0.718-1.277)	0.7682
≥4	157/369	98/209	0.5316	1.102 (0.813-1.494)	0.531	1.101 (0.812-1.495)	0.5355

^a^Adjusted for age.

## Data Availability

Data sharing is not applicable to this article because no datasets were generated or analyzed in the current study. Please contact the authors for data requests.
